# Comparison of Risk for End-Stage Renal Disease Between Physicians and the General Population: A Nationwide Population-Based Cohort Study

**DOI:** 10.3390/ijerph16122211

**Published:** 2019-06-22

**Authors:** Chin-Kai Yen, Tian-Hoe Tan, I-Jung Feng, Chung-Han Ho, Chien-Chin Hsu, Hung-Jung Lin, Jhi-Joung Wang, Chien-Cheng Huang

**Affiliations:** 1 Department of Emergency Medicine, Chi-Mei Medical Center, Tainan 710, Taiwan; i5488112@hotmail.com (C.-K.Y.); alloytan@gmail.com (T.-H.T.); nych2525@gmail.com (C.-C.H.); hjlin52@gmail.com (H.-J.L.); 2 Department of Medical Research, Chi-Mei Medical Center, Tainan 710, Taiwan; ixf34@case.edu (I.-J.F.); ho.c.hank@gmail.com (C.-H.H.); 400002@mail.chimei.org.tw (J.-J.W.); 3 Department of Hospital and Health Care Administration, Chia Nan University of Pharmacy and Science, Tainan 717, Taiwan; 4 Department of Biotechnology, Southern Taiwan University of Science and Technology, Tainan 710, Taiwan; 5 Department of Emergency Medicine, Taipei Medical University, Taipei 110, Taiwan; 6 Allied AI Biomed Center, Southern Taiwan University of Science and Technology, Tainan 710, Taiwan; 7 Department of Environmental and Occupational Health, College of Medicine, National Cheng Kung University, Tainan 701, Taiwan; 8 Department of Senior Services, Southern Taiwan University of Science and Technology, Tainan 710, Taiwan

**Keywords:** end-stage renal disease, hemodialysis, peritoneal dialysis, physician, renal transplantation

## Abstract

Physicians experience high stress and have much responsibility during a night shift, which contributes to increased sympathetic activity, the risk factor for renal disease. The risk for end-stage renal disease (ESRD) in physicians is still unclear. Therefore, we conducted a nationwide population-based cohort study to clarify this issue. Using Taiwan’s National Health Insurance Research Database, we identified 30,268 physicians and 60,536 individuals from the general population matched with a ratio of 1:2 by age and sex. All participants who had ESRD before 2006 and residents were excluded. ESRD risk between physicians and the general population and among physician subgroups was compared by following up their medical histories until 2012. We also compared the treatments between both cohorts with ESRD. Physicians had a lower ESRD risk than the general population (adjusted odds ratio (AOR): 0.5; 95% confidence interval (CI): 0.4–0.7), particularly in the middle-age subgroup (35–64 years) (AOR: 0.4; 95% CI: 0.3–0.7); however, there was no difference in the older age subgroup (≥65 years) (AOR: 1.0; 95% CI: 0.6–1.7). More physicians received peritoneal dialysis (63.0% vs. 11.1%) and renal transplantation (5.6% vs. 1.7%) than the general population after being diagnosed with ESRD. Compared with the general population, physicians had a lower ESRD risk and higher treatment selection for peritoneal dialysis and renal transplantation after being diagnosed with ESRD. Better medical knowledge, a greater awareness of diseases and their risk factors, more rigorous implementation of preventive measures, and easy access to medical care may play a role in this aspect. Further studies are warranted for elucidating the associated mechanisms.

## 1. Introduction

Chronic kidney disease (CKD) is an important worldwide problem for public health. The prevalence of end-stage renal disease (ESRD), the most severe form of CKD, is increasing worldwide [1]. According to the ESRD Medicare-funded program, the number of enrolled patients increased from 10,000 in 1973 to 615,899 in December 31, 2011, which is an increase of almost 60 times [2,3]. In 2012, Taiwan had the highest worldwide prevalence of ESRD patients receiving chronic dialysis (2902 per million population), which was about 20 times that in the country with the lowest prevalence [4].

Several risk factors have been proposed for CKD, including diabetes mellitus (DM), hypertension (HTN), hyperlipidemia, hyperuricemia, anemia, coronary artery disease (CAD), smoking, drugs, infections, family history, and urinary tract obstruction [5,6,7]. Risk factors for subsequent ESRD are higher levels of proteinuria, higher serum creatinine and uric acid levels, obesity, nonwhite race, lower education status, HTN, and DM [5]. Physicians are considered to hold a higher socioeconomic status than the general population. Privileged by better medical knowledge, optimal accessibility, and affordability and accountability of healthcare, physicians are believed to have a lower risk for acute myocardial infarction (AMI), sepsis, and cancer than the general population [8,9,10,11]. A study on the difference of dialysis use and survival between diabetic physicians and the diabetic general population suggested that an above-average medical knowledge of one’s own physical situation and more professional resources affect the outcome [12]. High work stress and night shifts are associated with increased sympathetic activity [6,13,14], which is a risk factor for renal disease [6,15,16,17,18]. A population-based study in the United States reported that health care practitioners had a higher risk for CKD, which may be related to their high work stress and night shifts [6]. Physicians in Taiwan have high work stress due to their long working time and high risk in medical practice [19,20]. A study reported that attending physicians in Taiwan had an average weekly work time of 65.6 h, which was much higher than that of the general population (43.7 h/week) [21]. Whether physicians have a lower or higher risk for ESRD than the general population is still in doubt. Therefore, we conducted this study to investigate the risk of ESRD in physicians in comparison with the general population.

## 2. Materials and Methods

### 2.1. Data Sources

We conducted this retrospective nationwide population-based cohort study using the Taiwan National Health Insurance Research Database (NHIRD). Taiwan launched a single-payer National Health Insurance program on 1 March 1995. As of 2014, 99.9% of Taiwan’s population was enrolled in the program [22]. Foreigners living in Taiwan are also eligible for this program. The database of this program contains registration files and original claim data for reimbursement. Large, computerized databases derived from this system by the National Health Insurance Administration, Ministry of Health and Welfare, Taiwan, and maintained by the National Health Research Institutes, Taiwan, are provided to scientists in Taiwan for research purposes.

### 2.2. Identification of the Study Cohort (Physicians) and Comparison Cohort (General Population)

**Figure 1:**
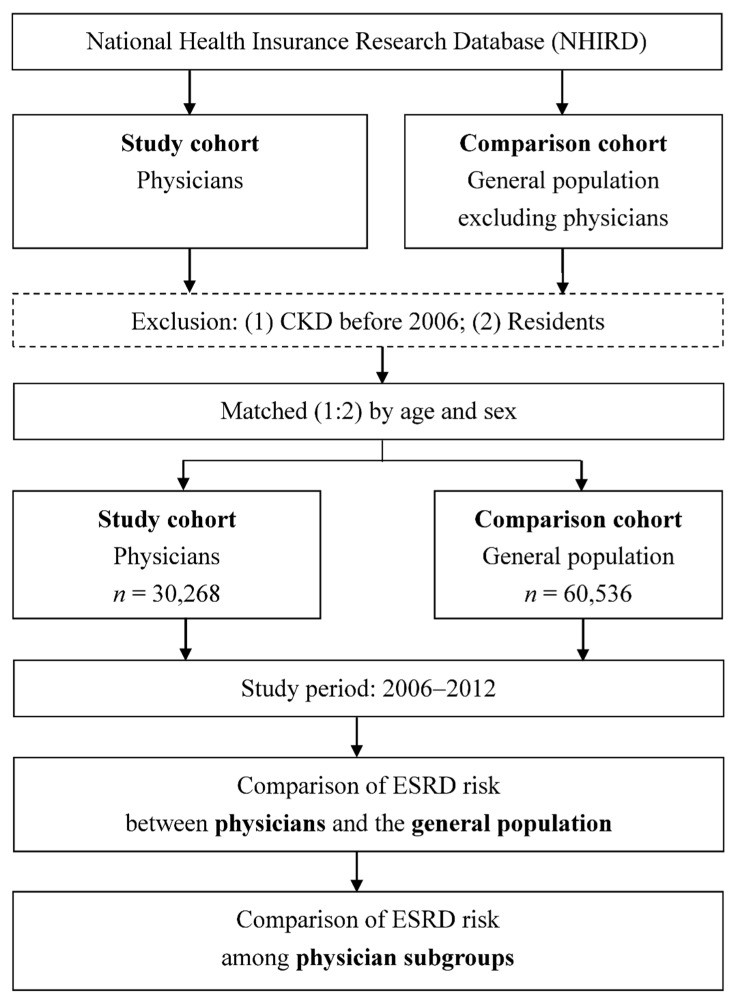
Flowchart of this study. LHID, Longitudinal Health Insurance Database; CKD, chronic kidney disease; ESRD, end-stage renal disease.

Using the NHIRD, we identified all physicians as the study cohort and the general population excluding physicians as the comparison cohort (Figure 1). Because we intended to investigate the effect of “being a physician” on the risk for ESRD, the physicians were set as the study cohort (exposed cohort) and the general population were set as the comparison cohort (unexposed cohort). After excluding the participants who had CKD (ICD-9-CM code 585) before 2006 or residents, physicians and the general population were matched by age and sex with a 1:2 ratio. Residents were excluded due to a very short exposure time in individual occupation. Comorbidities such as HTN (International Classification of Diseases, Ninth Revision, Clinical Modification (ICD-9-CM) code 401–405), DM (ICD-9-CM code 250), hyperlipidemia (ICD-9-CM code 272), hyperuricemia (ICD-9-CM code 274), anemia (ICD-9-CM code 280-285), and CAD (ICD-9-CM code 410-414) were recorded for both cohorts. The ICD-9-CM code diagnosis of the ESRD and comorbidities was made by the treating physicians. In order to avoid over-diagnosis by the ICD-9-CM code, the criteria for ESRD and comorbidities were defined as: (1) ≥1 time of diagnosis on admission or (2) ≥3 times of diagnosis on ambulatory care. The ESRD was also confirmed by the registry for catastrophic illness patients. Finally, 30,268 physicians and 60,536 individuals from the general population were identified for the study.

### 2.3. Comparison of ESRD Risk Between Physicians and the General Population and Among Physician Subgroups

We compared the ESRD risk between physicians and the general population and among physician subgroups by starting from 2006 and following up their medical histories until 2012 (Figure 1). Physician specialties were classified into internal medicine, surgery, obstetrics and gynecology (ob/gyn), pediatrics, emergency medicine, family medicine, and other specialties. Stratified analyses according to sex and age were also performed. We also compared the treatment choices and age at diagnosis and treatment, including peritoneal dialysis, hemodialysis, and renal transplantation, between physicians and the general population with ESRD.

### 2.4. Ethics Statement

This study was approved by the Institutional Review Board at Chi-Mei Medical Center. We conducted this study strictly according to the Declaration of Helsinki. Because the NHIRD contains de-identified information, informed consent from the participants is waived. This waiver does not affect the right and welfare of the participants.

### 2.5. Statistical Analysis

For the comparison of demographic characteristics, comorbidities, treatments for ESRD, and age at diagnosis and treatment for ESRD, we used an independent t-test for continuous variables and a chi-square test for categorical variables. For the comparison of ESRD risk between physicians and the general population, we used conditional logistic regression analysis by adjusting for HTN, DM, hyperlipidemia, hyperuricemia, anemia, and CAD. Stratified analyses by age subgroups (<35, 35–64, and ≥65 years) and sex (male and female) were also performed. Firth’s penalized likelihood approach logistic regression was used for a comparison of the two cohorts alive until the end of 2012. For the comparison among physician subgroups, we used unconditional logistic regression analysis by adjusting for age, sex, HTN, DM, hyperlipidemia, hyperuricemia, anemia, and CAD. SAS 9.3.1 for Windows (SAS Institute, Cary, NC, USA) was used for all analyses. 

## 3. Results

The mean age and proportion of male physicians were 46.7 ± 11.3 years (mean ± SD) and 86.4%, respectively (Table 1). Regarding age subgroups, physicians comprised 15.3%, 78.1%, and 6.6% in the <35, 35–64, and ≥65 year subgroups. In the comparison of comorbidities, physicians had a higher prevalence of HTN, hyperlipidemia, and hyperuricemia, but lower prevalence of DM and anemia than that in the general population.

The cumulative incidences of ESRD between 2006 and 2012 were 0.2% and 0.4% in the physician and comparison cohorts, respectively (Table 2). The comparison of ESRD risk showed that physicians had a lower ESRD risk than the general population by conditional logistic regression analysis with adjustment for HTN, DM, hyperlipidemia, hyperuricemia, anemia, and CAD (adjusted odds ratio (AOR): 0.5; 95% confidence interval (CI): 0.4–0.7). Stratified analysis by age showed that middle-age physicians had a lower ESRD risk than the middle-age general population (AOR: 0.4; 95% CI: 0.3–0.7). Younger-age physicians (<35 years) had a trend of a lower ESRD risk compared with the general population in the same age subgroup (AOR: 0.2; 95% CI: 0.03–2.0). Stratified analysis by sex showed that both sexes had a lower ESRD risk than that in the general population, but a comparison of female subgroups did not show any difference. After excluding the decreased participants, the result was similar to Table 2 (Table S1). 

There was no difference of ESRD risk among physician specialties and age subgroups (Table 3). Male physicians had a trend of a higher ESRD risk than female physicians. In the comparison of treatment choices in both cohorts with ESRD, a higher proportion of physicians received peritoneal dialysis and renal transplantation than the general population (63.0% vs. 11.1% and 5.6% vs. 1.7%, respectively) (Table 4). Physicians had an older age at first diagnosis of ESRD, first peritoneal dialysis, first hemodialysis, and renal transplantation than the general population (Table 5).

## 4. Discussion

This study showed that physicians had a lower ESRD risk than the general population. Stratified analysis by age revealed that middle-age physicians had a lower ESRD risk than the general population, whereas younger and older age physicians did not. Male physicians had a lower ESRD risk than the male general population, whereas the difference between female physicians and female general population was not significant. A higher proportion of physicians received peritoneal dialysis and renal transplantation than the general population. Physicians had an older age at first diagnosis of ESRD, first peritoneal dialysis, first hemodialysis, and renal transplantation than the general population. 

Better medical knowledge, a greater awareness of diseases and their risk factors, more rigorous implementation of preventive measures, and easy access to medical care may explain why physicians had a lower ESRD risk than the general population. In this study, physicians had a higher prevalence of risk factors for ESRD, including HTN, hyperlipidemia, and hyperuricemia, which suggested that physicians were not healthier than the general population, but have a better ability to avoid suffering from a more fatal disease by controlling their chronic comorbidities well. This result was compatible with previous studies on physician health in Taiwan [8,9,10]. A study on AMI in physicians showed that physicians had a higher prevalence of HTN and hyperlipidemia, but a lower risk of AMI, than the controls (AOR: 0.57; 95% CI: 0.46–0.72) [8]. Medical center physicians had a 50% AMI risk compared to local clinic physicians [8]. Another study showed that the risk of death due to sepsis was lower in physicians than in controls (adjusted hazard ratio, 0.82; 95% CI: 0.71–0.95) [9]. Physicians have a greater access to medical care and awareness of disease, which may permit timely treatment and prevent subsequent AMI and sepsis induced by delayed treatment [8,9]. The possible explanations for the lower prevalence of DM in the physicians than in the general population are better medical knowledge, higher disease awareness, and easier healthcare access in the physicians. Physicians may also have a lower body mass index and exhibit less obesity, which relate to a lower risk for DM. However, we could not confirm this association because the data of body mass index and obesity were not available in this study.

In the stratified analysis for age subgroups, younger-age physicians had a trend of a lower ESRD risk than the younger-age general population. Middle-age physicians had a lower ESRD risk than the middle-age general population; however, there was no difference in the older age subgroup. This suggests that when people age, the advantage of “being a physician” for ESRD risk decreases because of the increasing comorbidities [23,24]. Female physicians had a lower risk for ESRD than the female general population; however, the difference was not significant. The possible explanation for this is that the number of cases for ESRD is small (2 in female physicians vs. 14 in the female general population). Recruiting more participants in the future may help delineate this issue. 

In the comparison among physicians, older age was not an independent predictor for ESRD. Male physicians had a trend of a higher risk for ESRD than female physicians. There are no studies on renal disease in physicians. A study in Japan in 2007 showed that men had an ESRD incidence rate of 325 per million population, which was higher than the rate of 190 per million population in women [25]. Because of the advantage of medical knowledge and access to medical care, more physicians selected peritoneal dialysis and renal transplantation for the treatment of ESRD than the general population. The age at first peritoneal dialysis and age at renal transplantation were significantly older in the physicians than in the general population, which also suggests the stark difference between the two cohorts. Hemodialysis was the most common form of dialysis provided to ESRD patients [4]. The selection of peritoneal dialysis or hemodialysis is usually based on patient desire and motivation, convenience of access to hemodialysis, the treating physician, patient education, insurance, and the reimbursement system [26]. Patient satisfaction may be higher with peritoneal dialysis; however, peritoneal dialysis costs are significantly lower than those of hemodialysis [26]. If the reimbursement system includes the bundling of dialysis services, an increase in the number of patients on peritoneal dialysis can be expected [26]. 

Although this was a pilot study on ESRD in physicians, it had some limitations. First, we had no detailed information about smoking, drug use, drinking, obesity, body mass index, dietary, lifestyle, and region of residence such as rural versus urban in this study, which may be confounding factors. Third party data may be used to assess the effects of such confounders. However, we recorded the major risk factors for ESRD, including age, sex, HTN, DM, hyperuricemia, hyperlipidemia, anemia, and CAD. In addition, smoking and drug use are associated with the comorbidities we recorded. Therefore, the influence of other risk factors may be minimal. Second, we did not evaluate the possible environmental nephrotoxins and work schedule, including stress and night shifts among specialties, which made it difficult to interpret the results. Further studies on these factors and the associated mechanisms are warranted. Third, the study period (2006–2012) may be not sufficient. Therefore, a study with a longer period of follow-up may be needed to validate the results. Fourth, the estimation of survival analysis is a better method than logistic regression; however, the survival analysis needs to take into account the entry time of each observed participant. We did not know the exact time that physicians had been working in this position in the database we used, so we chose to use logistic regression for the comparison of ESRD risk between two cohorts. Fifth, this study has tended to reject the night shift- and work stress-related increased sympathetic activity hypothesis put forward as a likely driver of ESRD in physicians. However, we could not make the conclusion that night shift- and work stress-related increased sympathetic activity in the physicians did not affect the risk for ESRD because there was no measurement for night shifts and work stress in this study. Sixth, although this was a nationwide population-based study, the results may not be generalized to other nations due to the differences in race, diet, culture, and government policies.

## 5. Conclusions

This nationwide population-based cohort study showed that physicians had nearly half the ESRD risk of that of the general population. Better medical knowledge, a greater awareness of diseases, more rigorous implementation of preventive measures, and access to medical care may play major roles in this aspect. In the case of ESRD, the choice of modality in the physicians is in stark contrast to the general population. A higher number of physicians received peritoneal dialysis and renal transplantation than the general population, which reflects the importance of medical education for the selection of treatment. Further studies about the detailed associated mechanisms are warranted.

## Figures and Tables

**Table 1:** Demographic characteristics and comorbidities of the physician cohort and comparison cohort (general population).

Characteristic	Physician Cohort (*n* = 30,268)	Comparison Cohort (*n* = 60,536)	*p* Value	
Age (years)	46.7 (11.3)	46.7 (11.3)	0.957	
Age (years)			0.994	
Younger (<35)	4633 (15.3)	9249 (15.3)		
Middle (35–64)	23,630 (78.1)	47,274 (78.1)		
Older (≥65)	2005 (6.6)	4013 (6.6)		
Sex			>0.999	
Male	26,159 (86.4)	52,318 (86.4)		
Female	4109 (13.6)	8218 (13.6)		
Comorbidity				
HTN	8656 (28.6)	15,367 (25.4)	<0.001	
DM	5518 (18.2)	13,389 (22.1)	<0.001	
Hyperlipidemia	8603 (28.4)	11,912 (19.7)	<0.001	
Hyperuricemia	4003 (13.2)	7404 (12.2)	<0.001	
Anemia	1382 (4.6)	3507 (5.8)	<0.001	
CAD	2990 (9.9)	6018 (9.9)	0.775	

HTN, hypertension; DM, diabetes mellitus; CAD, coronary artery disease. *Data are expressed as number (%).

**Table 2:** Comparison of ESRD risk between the physician cohort and comparison cohort (general population) in relation to the overall group, different age subgroups, and sex using conditional logistic regression.

Characteristic	Number of ESRD (%)	OR (95% CI)	AOR (95% CI) *	*p* Value ^†^
Overall analysis				
Physician cohort	54 (0.2)	0.4 (0.3–0.6)	0.5 (0.4–0.7)	<0.001
Comparison cohort	243 (0.4)	1.0	1.0	
Stratified analysis				
Age subgroup				
Younger (<35 years)				
Physician cohort	1 (0.02)	0.199 (0.03–1.6)	0.2 (0.03–2.0)	0.180
Comparison cohort	10 (0.1)	1.0	1.0	
Middle (35–64 years)				
Physician cohort	33 (0.1)	0.3 (0.2–0.5)	0.4 (0.3–0.7)	<0.001
Comparison cohort	193 (0.4)	1.0	1.0	
Older (≥65 years)				
Physician cohort	20 (1.0)	1.0 (0.6–1.7)	1.0 (0.6–1.7)	0.958
Comparison cohort	40 (1.0)	1.0	1.0	
Sex				
Male				
Physician cohort	52 (0.2)	0.5 (0.3–0.6)	0.6 (0.4–0.8)	<0.001
Comparison cohort	229 (0.4)	1.0	1.0	
Female				
Physician cohort	2 (0.1)	0.3 (0.1–1.3)	0.4 (0.1–1.3)	0.115
Comparison cohort	14 (0.2)	1.0	1.0	

ESRD, end-stage renal disease; AOR, adjusted odds ratio; CI, confidence interval; HTN, hypertension; DM, diabetes mellitus; CAD, coronary artery disease. *Adjusted for HTN, DM, hyperlipidemia, anemia, hyperuricemia, anemia, and CAD. ^†^ For AOR.

**Table 3:** Comparison of ESRD risk among physician specialties by unconditional logistic regression.

	Number of ESRD (%)	OR (95% CI)	AOR (95% CI) *	*p* Value †
Specialty				
Internal medicine	9 (0.2)	1.5 (0.7–3.3)	1.3 (0.6–3.0)	0.512
Surgery	7 (0.3)	2.2 (0.9–5.1)	1.0 (0.4–2.4)	0.965
Ob/gyn	7 (0.3)	2.1 (0.9–4.9)	1.0 (0.4–2.4)	0.995
Pediatrics	4 (0.1)	0.9 (0.3–2.5)	0.9 (0.3–2.8)	0.853
Emergency medicine	0 (0.0)	–	–	–
Family medicine	6 (0.3)	1.8 (0.7–4.6)	0.7 (0.3–1.9)	0.493
Other specialties	2 (0.1)	1 (reference)	1 (reference)	–
Age subgroup (years)				
Younger (<35)	1 (0.02)	0.02 (0.003–0.2)	1.0 (0.1–8.7)	0.966
Middle (35–64)	33 (0.1)	0.1 (0.1–0.2)	1.1 (0.6–2.1)	0.760
Older (≥65)	21 (1.0)	1 (reference)	1 (reference)	–
Sex				
Male	52 (0.2)	4.1 (1.0–16.8)	3.3 (0.8–14.5)	0.109
Female	2 (0.1)	1 (reference)	1 (reference)	–

ESRD, end-stage renal disease; AOR, adjusted odds ratio; CI, confidence interval; Ob/gyn, obstetrics and gynecology; HTN, hypertension; DM, diabetes mellitus; CAD, coronary artery disease. * Adjusted for age, sex, HTN, DM, hyperlipidemia, hyperuricemia, anemia, and CAD. † For AOR.

**Table 4:** Comparison of treatment with peritoneal dialysis, hemodialysis, and renal transplantation in both cohorts with ESRD.

Treatment	Physician cohort with ESRD (*n* = 54)	Comparison cohort with ESRD (*n* = 243)	*p* Value
Peritoneal dialysis	34 (63.0%)	27 (11.1%)	< 0.001
Hemodialysis	10 (18.5%)	188 (77.4%)	
Renal transplantation	3 (5.6%)	4 (1.7%)	

ESRD, end-stage renal disease; OR, odds ratio; CI, confidence interval. *Data are expressed as number (%).

**Table 5:** Comparison of age at diagnosis or treatment in both cohorts with ESRD.

Age (years)	Physician cohort with ESRD (*n* = 54)	Comparison cohort with ESRD (*n* = 243)	*p-*Value
Age at first diagnosis of ESRD	61.21 ± 10.54	55.95 ± 11.23	0.002
Age at first peritoneal dialysis	61.44 ± 10.37	48.72 ± 10.38	<0.001
Age at first hemodialysis	59.16 ± 12.08	57.26 ± 10.84	0.526
Age at renal transplantation	54.61 ± 3.49	40.17 ± 8.60	0.037

ESRD, end-stage renal disease. * Data are expressed as mean ± standard deviation.

## Supplementary Materials

The following are available online at https://www.mdpi.com/1660-4601/16/12/2211/s1: Table S1: Comparison of ESRD risk between the physician cohort and comparison cohort (general population) in relation to overall groups, different age subgroups, and sex using Firth’s penalized likelihood approach logistic regression for the participants alive until the end of 2012.
