# Supplementary material for: Comparison of Risk for End-Stage Renal Disease Between Physicians and the General Population: A Nationwide Population-Based Cohort Study
Source: Int J Environ Res Public Health. 2019 Jun 22;16(12):2211. doi: 10.3390/ijerph16122211 (PMC6617167; doi:10.3390/ijerph16122211)
Supplement: Supplementary file 1 [file ijerph-16-02211-s001.pdf]

International Journal of
Environmental Research and Public Health

Table S1. Comparison of ESRD risk between physician cohort and comparison cohort (general population) among overall, different age subgroups, and sex using Firth's penalized likelihood approach logistic regression for the participants alive until the end of 2012.

	Number of ESRD (%)	OR (95% CI)	AOR (95% CI)*	*p* value†
Overall analysis				
Physician	51 (0.2)	0.5 (0.4–0.7)	0.5 (0.4–0.7)	<0.001
Comparison	207 (0.4)	1.0	1.0	
Stratified analysis				
Age subgroup				
Younger (<35 years)				
Physician	1 (0.02)	0.3 (0.03–1.4)	0.2 (0.03–1.1)	0.083
Comparison	9 (0.1)	1.0	1.0	
Middle (35–64 years)				
Physician	31 (0.1)	0.4 (0.2–0.5)	0.4 (0.3–0.6)	<0.001
Comparison	168 (0.4)	1.0	1.0	
Older (≥65 years)				
Physician	19 (1.0)	1.2 (0.7–2.2)	1.2 (0.7–2.2)	0.509
Comparison	30 (0.8)	1.0	1.0	
Sex				
Male				
Physician	49 (0.2)	0.5 (0.4–0.7)	0.5 (0.4–0.7)	<0.001
Comparison	197 (0.4)	1.0	1.0	
Female				
Physician	2 (0.05)	0.5 (0.1–1.6)	0.4 (0.1–1.4)	0.169
Comparison	10 (0.1)	1.0	1.0	

ESRD, end-stage renal disease; AOR: adjusted odds ratio; CI: confidence interval; HTN, hypertension; DM, diabetes mellitus; CAD, coronary artery disease. *Adjusted for age, sex, HTN, DM, hyperlipidemia, anemia, hyperuricemia, anemia, and CAD. †For AOR.
